# Arthroscopy-assisted reconstruction of coracoclavicular ligament by Endobutton fixation for treatment of acromioclavicular joint dislocation

**DOI:** 10.1007/s00402-014-2117-2

**Published:** 2014-11-25

**Authors:** Zhaoxun Pan, Hongxin Zhang, Chao Sun, Lianjun Qu, Yan Cui

**Affiliations:** Department of Joint Surgery, The Eighty-Ninth Hospital of People’s Liberation Army, Weifang, 261021 China

**Keywords:** Arthroscopy, Endobutton, Coracoclavicular ligament, Acromioclavicular dislocation, Ligament repair

## Abstract

**Objective:**

The aim of this study was to evaluate the clinical outcomes of arthroscopy-assisted reconstruction of the coracoclavicular (CC) ligament using Endobutton for treating acromioclavicular (AC) joint dislocation.

**Methods:**

From March 2012 to May 2013, a total of 22 patients with fresh AC joint dislocation (Rockwood type III and type V) were treated with arthroscopy-assisted Endobutton reconstruction of the CC ligament. The regular post-operation follow-up was performed. Shoulder joint function was assessed with Constant–Murley scores. Postoperative efficacy of the surgery was evaluated using the Karlsson criterion.

**Results:**

The 22 patients were followed postoperatively for an average of 24 months (16–31 months). Among them, 20 patients achieved good functional recovery with no pain. Two patients had slight pain in the acromion during shoulder joint motion with limited abduction at 3 months, both of whom had recovered at 6 months. Radiography confirmed anatomical reduction of the AC joint in all patients. At 1 year, the Constant–Murley scores were 93.1 ± 2.4 points on the injured side versus 94.2 ± 2.7 points on the uninjured side. The difference did not reach statistical significance (*P* > 0.05). Postoperative Karlsson evaluation ranked 20 patients (90.9 %) as grade A and 2 as grade B (9.1 %) at the 3-month follow-up. All patients had become grade A at 6 months. None of the patients had brachial plexus or peripheral vascular injuries.

**Conclusion:**

Arthroscopy-assisted reconstruction of the coracoclavicular ligament by Endobutton fixation is a safe, easy method for treating AC joint dislocation. It provides reliable fixation, causes little trauma, and has a fast recovery.

## Introduction

Acromioclavicular (AC) joint dislocation, caused by direct or indirect force, is frequently encountered injuries around the shoulder that accounts for 12 % of all shoulder joint injuries [1]. This kind of injury involves any age, and is a common injury in sports, military training, traffic accidents, and falls [2, 3]. The incidence of AC joint dislocation is high among professional athletes, recruits, and young patients [4–6]. The most regarded and useful classification is Rockwood’s one, from grades I to VI, which is based on the degree of injuries [7]. Grade I–II AC joint dislocations can be treated conservatively, while grades III (Special populations: for example, overhead athletes, heavy manual workers, etc.) and IV–VI generally require surgical intervention due to the common characteristics including both AC ligament and CC ligament disruption, instability in direction of horizontal and vertical [8, 9].

Although numerous surgical methods are used to treat AC joint dislocations, there is still no gold standard. These fixation methods have been linked with operation trauma, non-anatomic repair, and high incidence of postoperative complications [10–14].

To solve the above problems such as operation trauma and unsatisfactory recovery and to evaluate the clinical outcomes of arthroscopy-assisted reconstruction of the CC ligament using Endobutton for treating AC joint dislocation, arthroscopy-assisted Endobutton reconstruction of the CC ligament was accomplished in 22 patients with fresh AC joint dislocation (Rockwood type III–V) from March 2012 to May 2013.

## Materials and methods

### Patients

This clinical trial involved 22 patients, all of whom had a fresh traumatic dislocation of the AC joint. There were 16 male and 6 female patients. In all, 8 dislocations of the right joint and 14 of the left joint were treated. The patients were 17–44 years old (average 26 years). The duration between joint injury and surgical treatment was 2–14 days (average 6.1 days). The patients were classified as Rockwood types III (*n* = 15) and V (*n* = 7). Preoperative examination indicated the presence of a prominent, towering extremitas acromialis claviculae with deformity and displacement instability. Radiography showed that the coracoclavicular distance increased to about 100 % compared to the contralateral.

### Surgical procedure

**Fig. 1:**
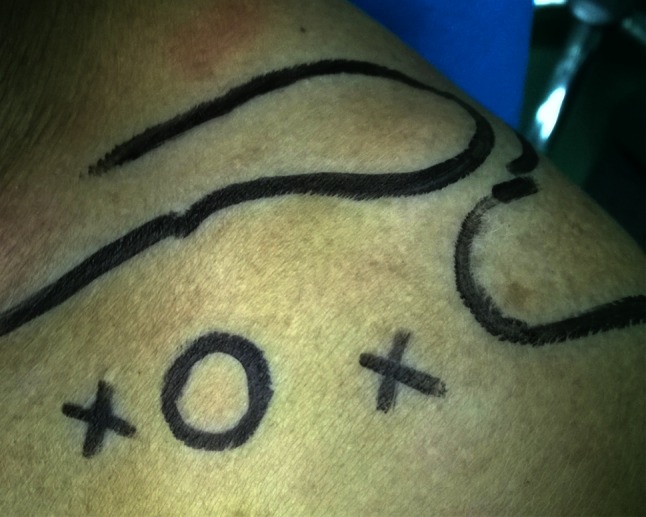
Arthroscopic portals: one was placed medial to the tip of the coracoid process and another was placed laterally or slightly inferior to the tip of the coracoid process

The surgery was performed on patients in a “beach chair” position under general anesthesia. Intraoperative systolic pressure was maintained at approximately 100 mmHg. Arthroscopic lavage fluid contained 1:100,000–1:300,000 epinephrine hydrochloride. The position of the lavage fluid was 80–100 cm above the surgical site. The positions of the clavicle, acromion, and coracoid process were marked on the body surface. Arthroscopic portals were selected as follows (Fig. 1): one was placed medial to the tip of the coracoid process (approximately 2.0 cm distance), and another was placed laterally or slightly inferior to the tip of the coracoid process (approximately 1.5 cm distance, generally the same as a standard anterior portal for the shoulder joint). The shoulder joint was explored internally through the lateral portal of the coracoid process or after establishing a standard posterior portal.

Epinephrine hydrochloride in saline (20 mL) was first injected for infiltration around the portals to the surface of the coracoid process. To establish the portals, a sharp incision (0.5 cm) was made at the mark, followed by blunt expansion toward the coracoid process using a pair of straight tongs. When approaching the coracoid process, a small detacher was used to establish a working space at the surface of the coracoid process.

**Fig. 2:**
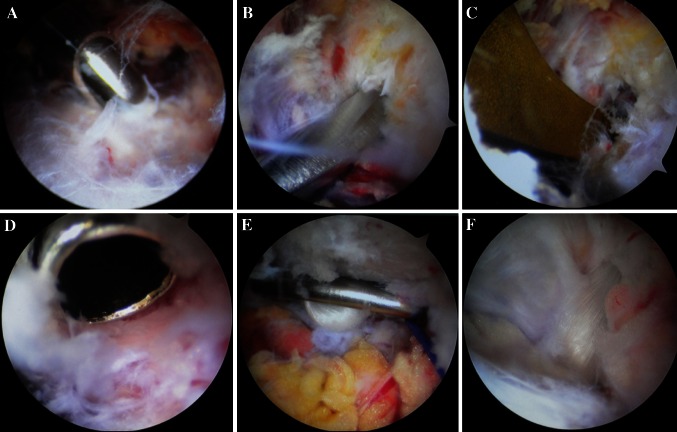
**a** The space around coracoid was cleaned to make a clear operation field and ensure an accurate identification of the orientation of the coracoid process. **b** A hand-held surgical detacher was used to strip closely along the distal surface of the coracoid process. **c** A knee joint posterior cruciate ligament locator was placed distally on the stripped surface at the base of the coracoid process. **d** Evaluated the surface of the coracoid process and inserted a guide device. **e** The Endobutton plate was overturned and placed across the dorsal surface of the coracoid process under arthroscopic control. **f** Observed the trapezoid fasciculi of the CC ligament

An arthroscope was placed at the portal lateral to the coracoid process, and surgical instruments were placed at the medial portal. The coracoid process was explored and identified under arthroscopic control. The working space was then expanded using the hand surgical detacher to obtain adequate room for performing the operation. The working space was then cleaned under arthroscopic control, and the orientation of the coracoid process and the directions of conoid and trapezoid fasciculi of the CC ligament were identified (Fig. 2a).

The coracoclavicular fascia was incised close to and along the inner edge of the coracoid process in the medial upper section using radiofrequency to expose the bone at the inner edge of the coracoid process. Through the incision, a hand-held surgical detacher was used to strip closely along the distal surface of the coracoid process (Fig. 2b). The stripped surface was positioned at the medial upper third of the coracoid process near the base (ligament insertion).

A 3- to 4-cm incision was made on the skin from the AC joint to the distal clavicle, which reached the periosteum. The AC joint and distal clavicle were exposed by subperiosteal separation. A knee joint posterior cruciate ligament locator was placed distally on the stripped surface at the base of the coracoid process under arthroscopic control (Figs. 1d, 2c). It was positioned proximally at the medioposterior third of the flat clavicle at the lateral end of the clavicle, 3 cm from the AC joint. A 2.0-mm K-wire was placed along a guide device, and three holes were drilled along the guide pin using a 4.5-mm core drill (denoted as hole A in the clavicle foramen and hole B in the coracoid foramen).

**Fig. 3:**
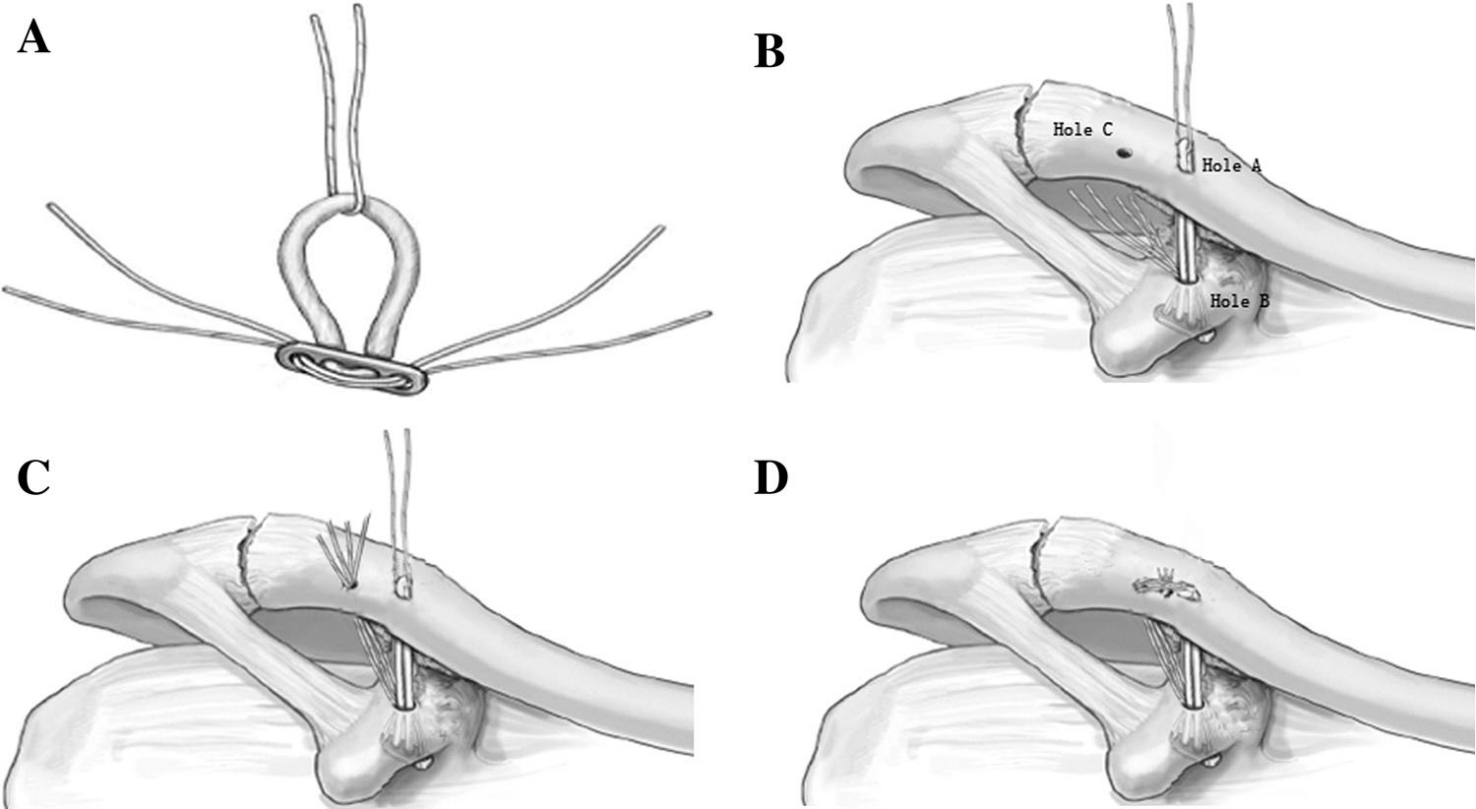
**a** Preparation of the implant. Two sutures have been passed through the holes of the Endobutton, One suture has been passed through the loop of the Endobutton. **b** The incision site and the place of the Endobutton plate. **c** Pulling methods of the three holes, *pilling lines* were pulled out through the surface of clavicle (hole **c**). **d** *Line* in hole **a** and **c** was strongly pulled, tightened, and then fixed by wrapping and knotting it in the surface of clavicle

The distance from the dorsal surface of the coronoid process to the superior surface of the clavicle was measured in the state of AC joint reduction. An appropriate length of Endobutton (available in 15- to 60-mm loop lengths (Smith & Nephew, Memphis, TN, USA) (Fig. 3a) was selected and used with a No. 5 Ethibond suture (Ethicon Inc., Somerville NJ, USA). According to the direction of the drill holes, a pulling line was introduced using a self-threading suture instrument. The pulling line (b) that was threading the loop of Endobutton and the fixing line (a) were pulled together through holes B and A. The Endobutton plate was placed across the dorsal surface of the coracoid process (Figs. 2e, 3b).

A hole lateral to the clavicle (denoted as hole C) was drilled using a 2.5-mm K-wire at the anterior medial third of the flat clavicle, 12 mm from the anterior lateral side of the clavicle foramen (hole A). Following the procedure mentioned above, a pulling line was threaded from hole C into the working space using a self-threading suture instrument. Line (a) was separated from the loop of the Endobutton under an arthroscope, and the two strands of line (a) were pulled out through hole C (Fig. 3c), followed by threading the loop of the Endobutton that had been exposed on the superior surface of the clavicle. The position of the Endobutton plate was adjusted under arthroscopic control to cross the dorsal surface of the coronoid process and tightly flattened against the bone surface. The AC joint was reduced and the distal clavicle lowered by 2–3 cm compared with the acromion. Line (a) was strongly pulled, tightened, and then fixed by wrapping and knotting it (Fig. 3d).

We ascertained that the AC joint was satisfactorily reduced and the Endobutton fixation was tight. Arthroscopically, we observed that the Endobutton loop crossed the medial section of the original CC ligament and extended next to the outer edge of the ligament (Fig. 2f) and that the two strands of line (a) crossed hole C and extended along the trapezoid fasciculi of the CC ligament. The torn trapezoid and conoid fasciculi of the CC ligament were reduced using a hook. In the case of a severe tear in the CC ligament (type V–IV), a suture was pre-set (prior to clavicular reduction) under arthroscopic control on the upper and lower ends of the torn ligament using a self-threading suture instrument. After reduction–fixation of the AC joint, the suture was knotted to enhance the repair. Hemostasis was then assessed, and the surgery was completed under arthroscopic control. Finally, the AC ligament was sutured, and the deltoid and trapezius fascial cuffs were repaired. The abovementioned tissues were sutured at the distal end of the clavicle. The incisions were cleaned and sutured.

### Postoperative treatment

After surgery, the injured arm was supported with a triangular sling for 1–2 weeks, during which time the patients began to practice aggressive circling with the injured shoulder. After discomfort ceased, the arm support was stopped, and the patients started a range of aggressive joint movement exercises. Up to 4 weeks postoperatively, the patients generally performed exercises that included only passive movement of shoulder joint. During weeks 4–6, the ligament was deemed to have healed sufficiently for the patient to practice progressively exercises with more resistance. From week 8, the patients began to return to normal activities and were allowed to start gentle throwing exercises. From week 12, the patients began to undertake heavy manual labor, confrontational exercises, and throwing training.

The joint range of motion was measured in both shoulders during the 1-year follow-up. To evaluate shoulder joint function, we obtained radiographs of the anterior and posterior positions of the AC joint at the 3-, 6-month and 1-year follow-up visits. Constant–Murley functional scores and Karlsson postoperative efficacy grades were also recorded.

### Criterion of postoperative efficacy

The Karlsson criterion [15] was used to rank the postoperative efficacy into three grades: A, no pain, normal muscle strength, free movement of shoulder, and anatomical reduction of the AC joint or subluxation within a 5-mm gap shown radiographically; B, satisfactory outcome, slight pain, restricted function, moderate muscle strength, shoulder joint range of motion in all directions >90°, and wider AC joint space on the injured side than on the uninjured side by 5–10 cm, shown radiographically; C, poor outcome, pain intensified at night, weak muscle strength, <90° range of motion in all directions for the shoulder joint, and AC joint dislocation shown radiographically.

### Statistical analysis

Statistical analysis of the 1-year Constant–Murley scores was performed using SPSS 18.0 software (IBM Inc., Armonk, NY, USA). Shoulder joint function was compared between the injured and uninjured sides using a paired *t* test, with *P* < 0.05 considered statistically significant.

## Results

### Postoperative physical examination

After the surgery, 22 patients were followed up for 16–31 months (mean 24 months). All patients achieved stage I healing of the incision, with no brachial plexus injury or peripheral vascular injury. During the 3-month follow-up, two patients had slight pain in the acromion during shoulder joint movement with limited abduction, although with strengthening exercises and symptomatic treatment they had achieved recovery at the 6-month follow-up. The other 20 patients had no shoulder discomfort, lassitude, or limited abduction. Radiography during the follow-up confirmed anatomical reduction of the AC joint in all patients.

### Results of Constant–Murley scores and Karlsson criterion

During the 1-year follow-up, the Constant–Murley scores were 93.1 ± 2.4 points on the injured side versus 94.2 ± 2.7 points on the uninjured side. The difference did not reach statistical significance (*P* > 0.05). Postoperative Karlsson evaluation ranked 20 patients (90.9 %) as grade A and 2 as grade B (9.1 %) at the 3-month follow-up. All patients had become grade A at 6 months. Shoulder joint function on the injured side recovered completely and maintained stability during the follow-up.

## Discussion

Nowadays various methods have been used to fix the AC joint; these methods use K-wires, screws, and clavicular hook plates. In the early days, K-wires were recommended for the fixation of AC joint by the Association for the Study of Internal Fixation (AO/ASIF), proposing that they were easy to operate, low cost and fixed firmly. However, they have been found to destroy articular surface and fibrocartilage, affect the clavicle rotation and limit the scope of lift on upper arms. In fact, studies have reported 25 % patients developed complications including calcification of the surrounding soft tissues and traumatic arthritis post-operation which were mainly attributed to the transarticular of the AC joint; it can also lead to pin-tail infection and K-wire loosening, breaking and prolapse [3], resulting in fixation failure; therefore, single application of K-wires has been eliminated. Fixation by compression screws between coracoids and clavicle is rigid fixation, which hampered the synchronous rotation of scapula and clavicle; in addition, screw loosening frequently occurred; therefore, now it is seldom used [16]. AO clavicular hook plate is a new fixation material developed a few years ago. It has the local anatomical features and biomechanical properties of AC joints, and is often used for the treatment of distal clavicle fractures and AC joint dislocations. Recently, however, various reports revealed many problems of this fixation method [17], such as unhook, impingement of the shoulder and shoulder joint pain, poor functional recovery and stress-induced fracture due to the lack of early exercise [18]. The use of clavicular hook plate hampered the repair of AC joint capsula and AC ligament and also neglects the repair of the CC ligament. Furthermore, remove of the clavicular hook plate often induced recurrence of the AC joint dislocation. In all, the above fixation methods are non-anatomic reconstruction which cannot restore the anatomic structure of the CC ligament and AC ligament, and the anatomical functions cannot fully restore though the above problems are avoided post-operation [19].

Struhl [20] reported good effect of the reconstruction of complete CC ligament using an Endobutton for the first time in 2007. He proposed that the procedure is simple, has certain effect, and can be adapted as a gold standard for the treatment of AC joint dislocation. With the increasing development of arthroscopic technique, the application field of arthroscopy has been a breakthrough. Undoubtedly, to overcome the technical problem and apply this technology to the complete arthroscopy operation is the best choice. Venjakob et al. [21] reported reliable stability of the AC joint in 96 % patients in a clinical research of arthroscopy-assisted double-Endobutton reconstruction of the AC joint with a Double-Tightrope technique for the treatment of AC joint dislocation and follow-up 58 months postoperatively. Natascha Kraus et al. [22] treated 15 patients (Rockwood type V) with arthroscopy-assisted operations, after a mean follow-up of 2 years; all patients were free of shoulder pain and movement restriction during daily activities. Shoulder function in affected side recovered to pre-injury level with an excellent and good rate of 96.2 %. Similarly, Markus Scheibel et al. [23] reported satisfactory results with an excellent and good rate of 95.1 % in a clinical study. Salzmann et al. [24] treated 22 patients (21 men, 2 women; mean age, 37.5 years) with arthroscopy-assisted operations with a mean follow-up of 30.6 months which reported a Constant–Murley score of 94.3 (range 88–98).

To date, continuous innovation and excellent surgical outcomes have been achieved with extra-articular arthroscopy [13]. In the present study, 22 patients with AC joint dislocation underwent arthroscopy-assisted surgery with a minimal incision at the distal end of the clavicle and a modified Endobutton procedure. This procedure does not rely on the length of the Endobutton to maintain the position, allowing accurate reduction of the AC joint. In previous reports, surgical methods to address AC joint dislocations involved a distance measurement after reducing the AC joint and drilling of the first tunnel, followed by selection of an Endobutton of appropriate length. The selection criterion for Endobutton length is that the loop can just go through the clavicular surface for fixation on and across the other Endobutton. However, the Endobutton loop is generally 15–60 mm long with 5-mm scales. There is the practical difficulty obtaining an Endobutton with a loop length that is exactly the same as the measurement made during surgery. Also, release of the temporal fixation inevitably causes rebound of the clavicle, which negatively affects joint reduction.

With our surgical method, Endobutton selection requires only that the loop length goes completely through the hole channels (A and B) and is exposed above the clavicular surface. Because the fixation is independent of the loop length, there is no strict requirement for the length of the loop to be exposed above the clavicular surface. In the circumstance of AC joint reduction, an exposed length of loop that is less than the distance between holes A and B is acceptable. By threading the Endobutton loop, the Ethibond suture threading hole C helps adjust the AC joint space to maintain good reduction and fixation of the injured joint using firm knotting. To enhance fixation strength, line (a), threading hole C, can be made up of two strands of Ethibond suture.

With our surgical method, anatomical specimens of the shoulder were examined before positioning drill holes A–C. In the CC ligament, conoid fasciculus appeared to be an inverted cone with the bottom attached to the tapered nodules at the lower posterior end of the clavicle and the tip to the medial margin at the base of the coracoid process. Lateral insertion of the clavicle was 3.0–3.5 cm from the distal end of the clavicle at the posterior third of the flat clavicle. Trapezoid fasciculus was located at the anterolateral side of the conoid fasciculus, with the upper end attached to the cristae obliqua on the superior surface of the clavicle and the lower end to the superior surface of the coronoid process. Lateral insertion of the clavicle was 1.6–2.0 cm from the distal end of the clavicle in the anterior third of the flat clavicle. Both conoid and trapezoid fasciculi were inclined in the medioinferior direction. The distance between the conoid and trapezoid fasciculi at the clavicle insertion edge was approximately 12 mm.

During surgery, hole B was positioned at the anterior edge of the coronoid insertion in the original ligament, close to the base of the coronoid process. Hole A was positioned at the medioposterior third of the flat clavicle and lateroanterior edge of the conoid fasciculus. Hole C was positioned at the inner edge of the trapezoid fasciculus, 12 mm from hole A at the anterolateral side. The drill holes were designed by taking into account bone strength and carrying capacity at the drilling sites, which approached the position of the original ligament. After Endobutton implantation and reduction–fixation with No. 5 Ethibond sutures, a solid triangular fixture was formed that meets the biomechanical requirements. The anatomical reconstruction of the CC ligament simulated the original ligament structure, thus ensuring stability of the AC joint in both the horizontal and vertical directions. The positioning of the drill holes in the bone avoided further injuries of the CC ligament by operations such as drilling, threading, and Endobutton implantation. Intraoperative reduction and suturing of the injured CC ligament provided favorable conditions for repair and healing of the original CC ligament. In addition, the AC ligament and deltoid and trapezius fascia cuffs were repaired and sutured through a small incision at the distal clavicle, thus restoring other stable structures of the AC joint and providing a comprehensive guarantee for full recovery and long-term stability.

Compared with Steven’s double-Endobutton fixation and triple-Endobutton fixation proposed by others, our single-Endobutton technique undoubtedly greatly reduces the cost of surgery. A combination of No. 5 Ethibond sutures (two or even four strands) and the Endobutton ensures sufficient mechanical strength for fixation. After surgery, the 22 patients had resumed normal activities by week 8 and began heavy manual labor and training during week 12. The postoperative follow-up (average 11 months; maximum 18 months) revealed no recurrent AC joint dislocation, and shoulder joint function was fully recovered.

Essential techniques of our surgical method and precautions for avoiding risks are summarized as follows:

1. Arthroscopic portals should be established correctly to provide an easy path to the coracoid process and proper working space. The medial and lateral portals are designed at a certain distance from the coracoid process to maintain an intact, nonleaking working space, so that an arthroscopy-assisted triangular three-dimensional operation can be easily accomplished.
2. The medioinferior position approximately 1 cm from the tip of the coracoid process includes the brachial plexus, subclavian artery, and musculocutaneous nerve, which are closest to the coracoid process. During preoperative establishment of arthroscopic portals and intraoperative operation of surgical instruments through the portals, direct access should follow the direction from the incision toward the anterior base of the coracoid process.
3. Operations at the coracoid process should be under arthroscopic control. A planer tool or radiofrequency can be used to remove floating fibrous tissues and stop bleeding for a clearer working space. During surgery, exposure of the medial section of the coracoid process and partial CC ligament insertion at the base of the coracoid process is sufficient. There is no need to completely expose or remove the inferior ligament insertion or the torn CC ligament to help the repair and healing.
4. The plane of the coracoid process is inclined from medioposterior to the lateroanterior direction relative to the chest wall. The medioinferior site 1 cm from the coracoid process includes the musculocutaneous nerve and the inferior brachial plexus and subclavian artery. However, the acromiocoracoid ligament and the tendons of coracobrachialis and biceps brachii are attached to the lateral surface of the coracoid process. Thus, we chose to incise the coracoclavicular fascia from the interior edge of the medial upper section of the coracoid process and strip it from the dorsal surface of the coracoid process for positioning and drilling of hole B.
5. When stripping the dorsal surface at the base of the coracoid process, the detacher should be handled closely against the bone surface to prevent loss of instrumental control and subsequent injury to the brachial plexus and important blood vessels.
6. Drilling holes in bone is assisted by a posterior cruciate ligament locator, whose wide front end can block both the K-wire and the drill. Alternatively, a depth control device can be used when drilling for the K-wire and core drilling. The selected K-wire should have the same diameter as the guide device to avoid wire deviation with a smaller diameter.
7. During introduction of the Endobutton with a self-threading suture instrument, the operation next to the coracoid process should be arthroscopy assisted.
8. If AC joint dislocation is associated with cartilage disk injury of the joint, the injured tissues should be resected during surgery to avoid postoperative pain and discomfort.
9. It is not recommended that the injured AC joint be fixed with temporary K-wire fixation because of the risk of long-term traumatic arthritis. Instead, it is recommended that the AC joint be reduced by hand pressure, making the distal clavicle lower by 2–3 cm, followed by knotting and fixation.
10. The CC ligament with a scattered tear should be identified and reduced. For a serious tear, a suture can be pre-set, before Endobutton fixation, using a self-threading suture instrument and then knotted and fixed after Endobutton fixation.

## Conclusion

Arthroscopy-assisted reconstruction of the CC ligament by Endobutton fixation achieved good clinical outcomes in patients with AC joint dislocation. This surgical method provides an ideal approach for clinical treatment of AC joint dislocations. It has a variety of advantages, including easy operation, little trauma, low cost, reliable effectiveness, fewer complications, and no need for a second surgery. Because of the relatively short follow-up in our study, large-sample clinical trials with long-term follow-up are needed to examine the long-term effectiveness of the proposed method.
